# Glioblastoma Treatment Modalities besides Surgery

**DOI:** 10.7150/jca.32475

**Published:** 2019-08-27

**Authors:** Hao Zhang, Ruizhe Wang, Yuanqiang Yu, Jinfang Liu, Tianmeng Luo, Fan Fan

**Affiliations:** 1 Department of Neurosurgery, Xiangya Hospital, Central South University, Changsha, Hunan Province, China; 2 Department of Urology, Xiangya Hospital, Central South University, Changsha, Hunan Province, China; 3 Department of Medical Affairs, Xiangya Hospital, Central South University, Chang Sha, Hunan Province, China; 4 Center for Medical Genetics & Hunan Provincial Key Laboratory of Medical Genetics, School of Life Sciences, Central South University Changsha, China

**Keywords:** glioblastoma, novel treatment, therapy

## Abstract

Glioblastoma multiforme (GBM) is commonly known as the most aggressive primary CNS tumor in adults. The mean survival of it is 14 to 15 months, following the standard therapy from surgery, chemotherapy, to radiotherapy. Efforts in recent decades have brought many novel therapies to light, however, with limitations. In this paper, authors reviewed current treatments for GBM besides surgery. In the past decades, only radiotherapy, temozolomide (TMZ), and tumor treating field (TTF) were approved by FDA. Though promising in preclinical experiments, therapeutic effects of other novel treatments including BNCT, anti-angiogenic therapy, immunotherapy, epigenetic therapy, oncolytic virus therapy, and gene therapy are still either uncertain or discouraging in clinical results. In this review, we went through current clinical trials, underlying causes, and future therapy designs to present neurosurgeons and researchers a sketch of this field.

## Introduction

Glioblastoma (GBM) is one of the most challenging tumors for physicians in oncology field 1-3. While many researchers devoted to exploring potential treatments through its molecular mechanisms 4-8, surgery remains to be the first choice for tumor de-bulking and accessing tissue sample for pathology. As long as neurological functions not compromised, maximal tumor resection may be beneficial 9. However, surgery alone is never enough. Because of the infiltrative nature of GBM, surgical resection alone leads to median survivals of only 3 to 6 months 10. In the past 60 years, with the development of radiotherapy, postoperative survival has been significantly improved to approximately one year by adding adjuvant radiotherapy alone^11^. Currently, concomitant radiation and oral alkylating agent temozolomide (TMZ) extend survival to 14 to 16 months 12. In this paper, we review current therapies other than surgery to provide a scheme for neurosurgeons and other researchers of interest (Table 1).

## 1. Chemotherapy

### 1.1. Temozolomide

**Figure 1:**
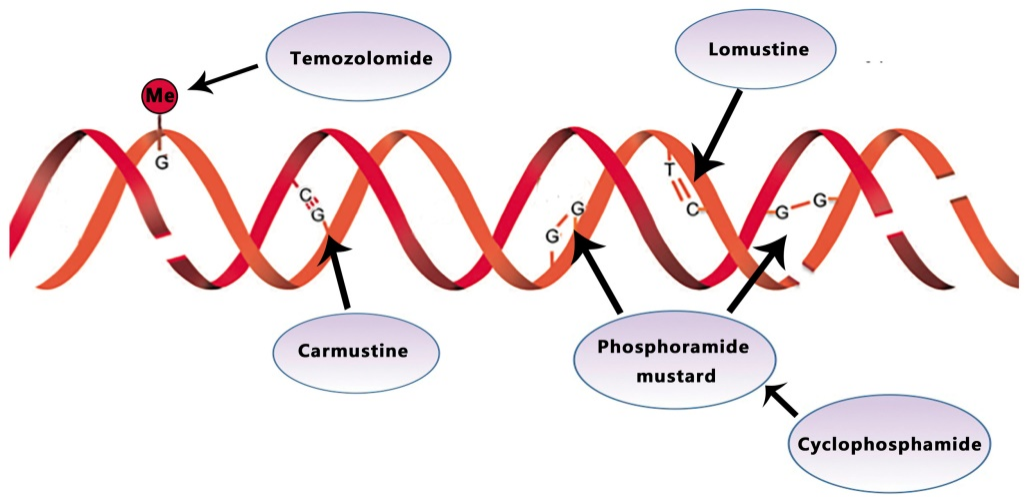
Temozolomide, lomustine, carmustine, and cyclophosphamide inhibit the tumor growth by alkylating/methylating DNAs and impeding DNA crosslinking

Temozolomide (TMZ) is an orally active alkylating agent approved by the United States Food and Drug Administration (FDA) to treat newly diagnosed GBM in March 2005. The therapeutic benefit of temozolomide depends on its ability to alkylate/methylate DNA, which most often occurs at the N-7 or O-6 positions of guanine residues (Fig. 1). Concomitant adjuvant TMZ chemotherapy and radiotherapy is the current standard of care for GBM patients 13. In a large randomized trial, TMZ combined with radiation significantly improved (TMZ/RT →TMZ) median, 2- and 5- year survival for GBM patients with a median OS of 14.6 months (compared with 12.1 months using radiotherapy alone) 14. As recommended in ESMO guideline, TMZ is administered daily (7 days a week) during radiotherapy for 5 days every 4 weeks for six cycles after radiation 13. A Phase III Clinical Trial demonstrates no improved efficacy for DD (dose-dense) temozolomide (days 1 through 21 of a 28-day cycle) for newly diagnosed GBM (NCT00304031) 12.

(O6-methylguanine-DNA methyl-transferase, MGMT), a DNA repair enzyme, plays a significant role in TMZ resistance. MGMT promoter methylation was found in approximately 45% of GBM. By silencing the gene on the epigenetic level, MGMT methylation decreased tumors' DNA repair capacity, increasing temozolomide susceptibility 15. For patients without MGMT promoter methylation, O6-benzylguanine, another inhibitor of MGMT, and RNA interference-mediated MGMT silencing offer promising avenues to increase TMZ efficacy 16. MGMT methylated tumors were associated with improved OS (21.2 v 14 months) and PFS (8.7 v 5.7 months) compared with un-methylated ones 12.

### 1.2. BCNU

BCNU (carmustine)-polymer wafers (Gliadel) were nitrosoureas approved by the FDA in 2002. As an alkylating agent, carmustine forms inter-strand crosslinks in DNAs to prevent DNAs from replication or transcription (Fig. 1). For newly diagnosed GBM, one recent research shows that Carmustine-impregnated wafers significantly improve survival 17. However, Gliadel wafers also were reported to associate with increased cerebrospinal fluid (CSF) leakage and increased intracranial pressure due to brain edema, despite the moderate increase in median survival (13.8 vs. 11.6) 18. In recurrent GBM, Jungk C proves that BCNU was rarely associated with severe side effects, particularly pulmonary toxicity and conferred favorable outcomes 19.

### 1.3. Lomustine

Lomustine is an alkylating nitrosoureas compound that influences DNA crosslinking and causes the methylation of the amino group (Fig. 1). It is particularly effective to treat tumors of the central nervous system due to its high lipid solubility to penetrate the BBB (blood-brain barrier) 20, 21.

The median OS after lomustine treatment is 9.1 months in primary glioblastoma 22 compared with the median OS of 7.5 months in recurrent glioblastoma 23 according to a phase II study (NCT01562197). In a recent phase III trial (NCT01149109), the median OS was significantly improved from 31.4 months with temozolomide alone to 48.1 months combining with lomustine-temozolomide 24.

Besides, lomustine was used as a comparator. In the first phase III trial (2005-004627-18) conducted in patients with recurrent glioblastoma, kinase C inhibitor Enzastaurin failed to demonstrate superior PFS to lomustine (6-month PFS 11.1% vs 19.0%) 25. A subsequent phase III monotherapy trial in the same patient selection (2007-000383-24) also demonstrate few significant PFS improvement with oral cediranib, which supposedly functioned as pan-vascular endothelial growth factor (pan-VEGF) receptor tyrosine kinase inhibitor^26^.

### 1.4. Cyclophosphamide

Cyclophosphamide (CPA) works against tumor through its metabolite phosphoramide mustard. This metabolite can alkylate DNA and form a cross connection to affect DNA function, thereby inhibiting the growth and reproduction of tumor cells and giving play to its anticancer effect (Fig. 1). In phase II studies of CPA, one with recurrent temozolomide-refractory glioblastoma showed 6-month PFS of 20%, the other with anaplastic astrocytoma showed 30% respectively^27^, ^28^.

One recent research suggested that CPA improves survival in orthotopic GL261 GBM in mice by metronomic administration (every 6 days) 29, and similar results were then described by several other studies 30, 31. So, more researches are guaranteed to futher evaluate the potential effect of CPA.

## 2. Radiotherapy

The post-surgery care standard for patients 70 years old or younger is partial-brain fractionated radiotherapy with concomitant TMZ. For better treatment, radiation should start as soon as safety assured. In clinical trials, researchers typically initiate radiation in 3 to 6 weeks after surgery 32. Despite the infiltrative nature of GBM, partial-brain radiation, recommended in the guideline, leads to no worse survival than whole-brain radiotherapy.

For patients under 70 years old, optimal dose fractionation schedule for EBRT (external beam radiation) after surgery is 60 Gy in 2-Gy fractions administered over 6 weeks 32, while other plans have not provided more benefits. Two early reports demonstrated no benefit in treating GBM with EBRT when doses exceeded 60Gy in standard fractionation 33, 34. As a result, most subsequent studies focused on doses equal to or less than 60Gy.

For patients over 70 years old, HFRT (hypofractionated radiotherapy) is recommended. Several recent studies suggested that the median OS of patients with HFRT was increased to 20 months, compared with conventional RT 35-40. HFRT benefit patients in limiting tumor repopulation, increasing cell kill, and reducing the overall treatment time 41, 42.

A prospective study with 60 Gy delivered in 20 fractions demonstrated an OS of 9.5 months with a 1-year OS of 40 % and a PFS of 5.2 months under the condition that post-operative tumor volume <110 cm^3^, and acute toxicity in this study was limited to brain edema in 2 patients 43. More recently, in a phase II trial(NCT01702610) TMZ was administered for 2 weeks before HFRT of 60 Gy in 20 fractions with concurrent and adjuvant TMZ. Results in this trial revealed that the median OS and PFS were 22.3 months and 13.7 months respectively 44. Besides, The University of Colorado trials (NCT01209442) delivered a dose of 60 Gy in 10 fractions with concomitant and adjuvant TMZ and BEV and reported a median OS of 16.3 months 45 with RN (radio-necrosis) in 50% patients.

Other commonly used hypo-fractionations include 40Gy in 15 fractions and 25Gy in 5 fractions. However, 25Gy in 5 fractions showed non-inferiority to 40Gy in 15 fractions (7.9 vs 6.4 months) 46 in a phase III trial (NCT01450449), and 40Gy in 15 fractions presented non-inferiority to 60Gy in 30 fractions (5.6 vs. 5.1 months) 47.

Although HFRT is associated with RN, patients who developed RN had improved survival compared with those who did not 40, 45, 48, and their quality of life was not affected by RN 49. Some studies supported that the use of Bevacizumab can potentially decrease the risk of RN 45, 50 or even treat RN 51.

In sum, the best practice of HFRT is still unclear, and further studies on HFRT are needed to better balance doses, fractions, RN, and life quality of patients.

## 3. Tumor treating field

**Figure 2:**
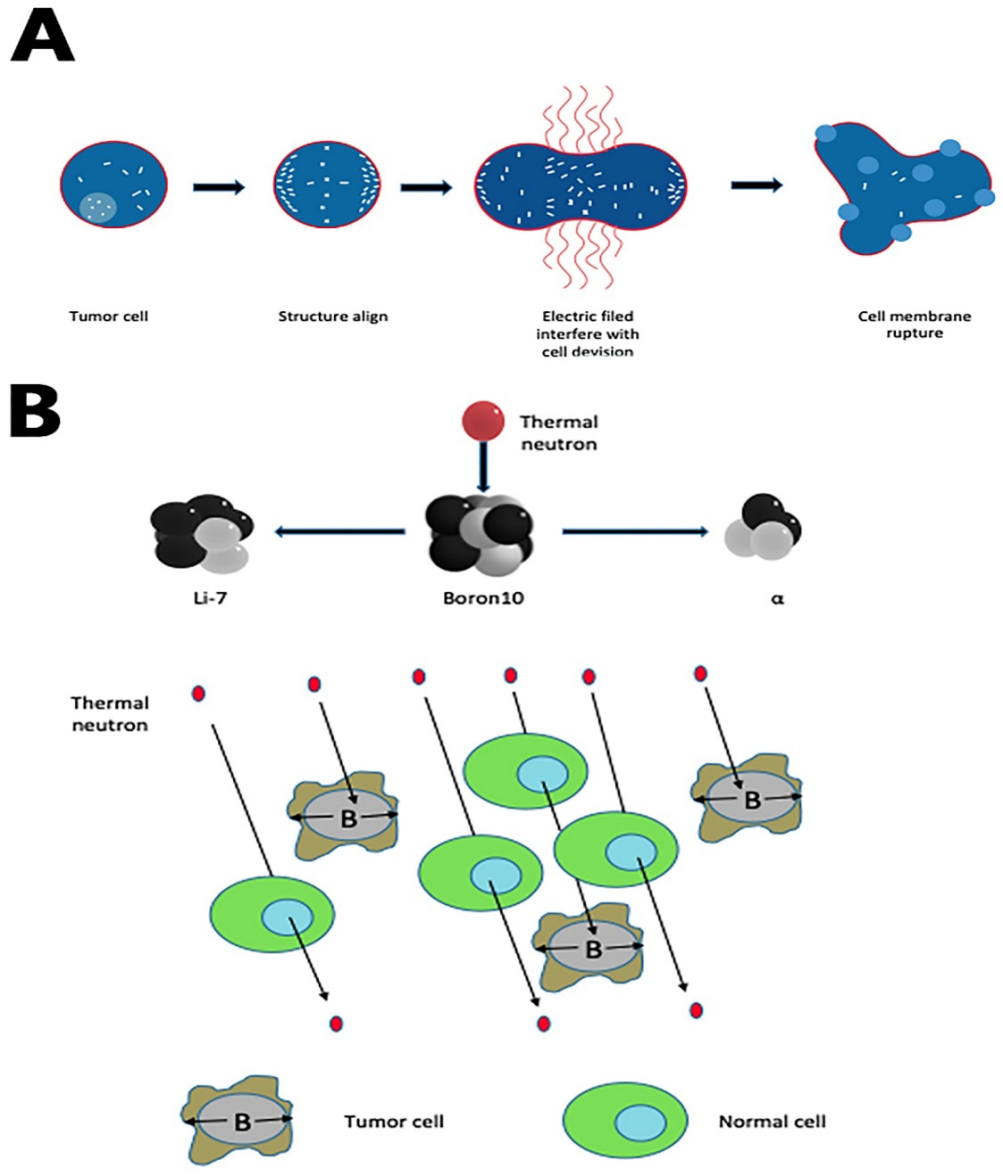
(A) TTF rupture tumor cell membrane by accumulating all polar molecules and dipoles in the cleavage furrow during the interdivision stage. (B) Boron-10 in tumor cells, radiated with thermal neutrons, releases high linear energy transfer (LET) α and 7Li particles. Both alpha particles and the lithium ions produce closely spaced ionizations in the immediate vicinity of the reaction, leading to a selective tumor cell killing.

Tumor treating fields (TT-Fields) are a new modality to treat newly diagnosed and recurrent GBM. Optune, a portable medical device, uses low-intensity, intermediate- frequency, alternating electric fields (Tumor-treating filed, TTF) to disturb highly orchestrated dividing processes in GBM cells, sparing quiescent ones 52-54. The mechanism of TT-Fields has two fundamental steps:(1) during the formation of mitotic spindles, the microtubule assembly deforms; mitosis of tumor cells remains in the interdivision stage for a long time 55; (2) when cleavage furrow forms in mid to late mitosis, all polar molecules and dipoles in cells undergo di-electrophoresis under the action of TT-Fields, which accumulates in the cleavage furrow and eventually causes the cell membrane to rupture^54^ (Fig. 2A).

TTFs are generated via electrodes on the scalp with unique array placement based on individual's MRI results. Optune, or the NovoTTF-100A System, was approved by FDA to treat recurrent GBM in 2011 and for newly diagnosed GBM in October 2015 56. The decision was based on a 700 patient phase 3 clinical trial, in which patients treated with TMZ plus standard treatment was associated with a median OS and PFS of 19.4 and 7.1 months respectively, compared to 16.6 (p=0.0222) and 4.2 months (p=0.0010) for those had only standard treatment 57. Those results were reported at ASCO 2015, and experts concluded that TT-Fields should be considered for GBM patients if not contraindicated.

Also, one recent phase 3 clinical trial (NCT00916409) demonstrates that health-related quality of life (HRQoL), cognitive, and functional status is not adversely affected by the continuous use of TT-Fields 58. Median OS in the TT-Fields plus chemotherapy group was significantly longer versus chemotherapy alone (11.8 vs. 9.2 months)^59^, supporting the therapeutic effect of TT-Fields.

## 4. Boron neutron capture therapy (BNCT)

Boron neutron capture therapy depends on the nuclear capture and fission reactions after boron-10 radiated with thermal neutrons from nuclear reactors. This reaction produces high linear energy transfer alpha particles (He-4) and lithium-7 since these alpha particles have a shorter path-length in tissues (5-9μm) than tumor cell diameters (about 10μm). In theory, if we use boron compound to deliver boron-10 precisely to tumor cells, this reaction can lead a selective tumor cell killing without damaging adjacent healthy cells (Fig. 2B). In the past decades, as the new boron compound boronophenylalanine (BPA) is designed, epithermal neutron beam introduced, and the proper computational system created, BNCT has evolved from intraoperative BNCT (IO-BNCT) to non-operative BNCT (NO-BNCT)^60^. The results from pre-clinic studies are prospecting. One recent study found a new dual-targeting strategy using c(RGDyC)-LP to improve BNCT for glioblastoma 61. Yoshiya Iguchi et al. created a compound called BSH-3R, which can efficiently increase boron uptake in cells 62.

In a Japanese clinical trial (NCT00974987) of 23 patients with newly diagnosed GBM, BNCT alone after surgery provided a mean survival time of 19.5 months 63. In a Swedish clinical trial of 29 newly diagnosed GBM patients, the mean survival time of BNCT alone group was 17.7 months compared to 15.5 months in the standard treatment group 64. In both trials, no adjuvant TMZ therapy or radiotherapy was added to BNCT therapy; hence, researchers had initiated phase II clinical trials of combining radiation and TMZ with BNCT to treat newly diagnosed GBM patients. However, designing more tumor-selective boron compound and replacing nuclear reactors with particle accelerators remain challenging.

## 5. Anti-angiogenic therapy

### 5.1. Bevacizumab

**Figure 3:**
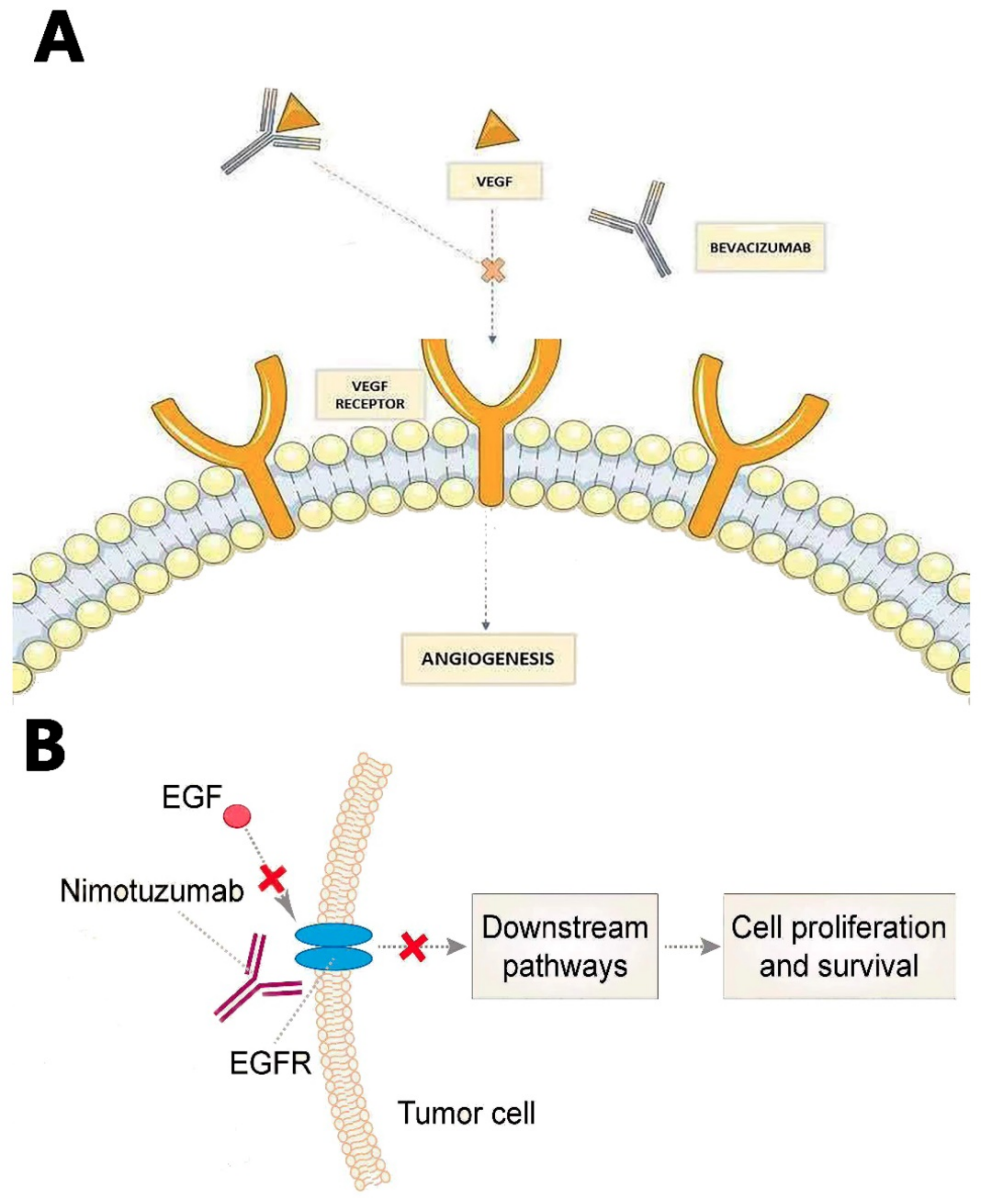
(A) Bevacizumab prevents VEGF from binding its receptor on endothelial cells to reduce tumor angiogenesis and tumor growth. (B) Nimotuzumab binds to EGFR, blocking consequential downstream pathways to inhibit angiogenesie and GBM growth

Recurrent GBMs were also treated with Bevacizumab, which is an IgG1 humanized monoclonal antibody that binds to VEGF ligand (Fig. 3A). It was approved by FDA in 2009 based on the success of two Phase II clinical trials 65, 66. Bevacizumab inhibits VEGF to bind its receptor flt-1 and KDR on endothelial cells to reduce tumor angiogenesis and tumor growth by inactivating VEGF. Since angiogenesis is inhibited by Bevacizumab, BBB will be less disrupted. Therefore, patients may show excellent radio-graphics such as decreasing tumor enhancement and FLAIR (fast fluid-attenuated inversion recovery) hyper-intensity. However, most patients will have non-enhancing tumor progress after 3-5 months 67. Some researchers believe Bevacizumab may increase the incidence of distant and diffuse tumor recurrence, turning GBM to a more aggressive phenotype; however, there is no convincing evidence on this debate 68.

In a recent clinical trial (NCT00884741), for newly diagnosed glioblastoma, duration of median OS between the Bevacizumab group and the placebo group is 15.7 and 16.1 months respectively, which showed no significant difference. Nevertheless, the median PFS was longer in the Bevacizumab group (10.7 months vs. 7.3 months) 69. One recent research recommended the use of Bevacizumab to prolong PFS and OS in the recurrent setting either alone or in combination with a cytotoxic agent, yet not in the primary setting. Unfortunately, the absolute survival advantage is limited to 4 months 70.

For newly diagnosed glioblastoma, adding Bevacizumab to standard therapy does not improve overall survival and is associated with a higher chance of early adverse events such as hypertension, thromboembolic events, etc. 71. Bevacizumab may be beneficial in prolonging progression-free survival, but still, Bevacizumab's routine addition to standard therapy for newly diagnosed GBM is not recommended in clinical practice 13.

### 5.2. Nimotuzumab

Nimotuzumab, Nimotuzumab is a humanized monoclonal antibody that binds to epidermal growth factor receptor (EGFR) and alters cell division. EGFR belongs to the ErbB family, which is related to many downstream pathways whose mutation or activation would facilitate angiogenesis and GBM growth 72 (Fig. 3B).

A phase II trial provided evidence of improved median OS in patients with high-grade glioma when treated with Nimotuzumab and RT compared to RT alone (17.8 vs12.6 months) 73. But the results are not directly applicable to GBM patients because most patients in this trial have anaplastic astrocytoma other than GBM. A German phase III trial (NCT00753246) showed no significant PFS or OS improvement in GBM patients treated with standard chemo-radiation (temozolomide and RT) with or without nimotuzumab 74. Interestingly, Nimotuzumab showed a trend of improved efficacy when administered to MGMT non-methylated GBM patients in both studies.

In a recent study conducted in Chinese patients, the median PFS and OS of Nimotuzumab in combination with TMZ and RT were 10.0 and 15.9 months, respectively, showing favorable safety and tolerability profiles in newly diagnosed GBM 75.

Whether Nimotuzumab should be used as front-line therapy was still debated, more researches are needed.

### 5.3. Depatuxizumab mafodotin

Depatuxizumab mafodotin (ABT-414) is monoclonal antibody-drug conjugate that binds to EGFR amplified on GBM and works against tumor through EGFR blockage and tubulin polymerization by conjugated tubulin inhibitor monomethyl auristatin F. Recently, ABT-414 demonstrated a median OS of 10.7 months in recurrent GBM patients 76. Besides, a subsequent phase I study (NCT01800695) proved the efficacy and safety of ABT-414 and temozolomide in recurrent GBM patients with a median OS of 7.4 months 77. Further studies are ongoing to evaluate its efficacy in newly diagnosed (NCT02573324) and recurrent glioblastoma (NCT02343406).

## 6. Immunotherapy

The brain is less immune privileged as researchers once thought; however, it is still immunologically specialized and provides tumors a sanctuary from systemic immunotherapy chemotherapy 78. Current immunotherapy for brain tumors can be categorized into vaccination, adoptive cell therapy, checkpoint inhibition, and immunostimulant 79. Open clinical trials of immunotherapy mainly focus on DC (dendritic cell) vaccination and antibodies aiming at checkpoint inhibitors, with promising, but not durable or sustainable clinical responses 80.

### 6.1. Vaccination

In 2008, PEP-3-KLH vaccine was first reported to induce newly diagnosed GBM patients to produce an EGFRvIII-specific antibody 81.EGFRvIII (type III epidermal growth factor receptor mutation), which is present in 24-67% of patients with GBM, allowing the continuous activation of EGFR 82.

Although some phase I/II clinical trials about PEP-3-KLH vaccine have encouraging results 83, the failure of phase III clinical trial (NCT02546102) investigating PEP-3-KLH vaccine set back the prospects for EGFRvIII-targeted peptide vaccine 84.

### 6.2. Adoptive cell therapy

Introducing chimeric antigen receptors (CARs) into T cells (CAR-T) to generate tumor-specific T cells, targeting an ideal marker EGFRvIII 85, has been the most widely-used ACT approach.

Phase I and II clinical trials have demonstrated significantly higher PFS and OS (26 months vs. 14.6 months) in vaccinated patients with EGFRvIII-expressing GBM tumors 86. The accuracy of ACT has demonstrated great potential in treating GBM. Two clinical trials, (NCT02209376) and (NCT01454596) are underway, expected to have positive outcomes.

### 6.3. Check-point inhibition

The agents act by blocking the immunosuppressive check-points that inhibit cytotoxic T cells, leading to intensified anti-tumor immune responses. In this field, PD-1/PDL1 pathway is drawing most attention of researchers 87. PD-1 reduces T-cell activity, inducing tolerance, and decreasing autoimmunity. One of its ligand, PD-L1, is highly expressed in GBM 88. In a preclinical study using GL261 glioma mouse model, anti-PD-1 therapy combined with radiotherapy doubled median survival in 15-40% of mice compared with either treatment alone^89^. This promising strategy is recently supported by a randomized clinical trial, in which anti-PD-1 immunotherapy provided significant survival benefits (median OS=13.7 months, PFS=3.3months) 90.

Another pathway, CTLA-4, was also associated with improved survival when used to enhance tumor-lysate vaccines in GBM mouse model 91.

### 6.4. Immunostimulant

Though associated with significant CNS toxicity 92-94, IL-2, the best studied Th1 cytokine for GBM, has been frequently used in vitro to activate lymphokine-activated killer cells (LAK) with broad-spectrum antitumor effect. One encouraging report of IL-2 treatment in recurrent GBM was with the median OS of 12.2 months 95. 75% 1-year survival in GBM 96 and 34% 1-year survival in recurrent GBM 97 shows the possible clinical benefits of using LAK stimulated by IL-2. One recently completed clinical trial (NCT01144247) of low dose IL-2 in the treatment of recurrent GBM has not been reported yet.

Although promising, immunotherapy can induce a high frequency of immune-related adverse effects that underlie the need for non-immunosuppressive and/or anti-inflammatory approaches 98.

## 7. Epigenetic therapy

Epigenetic changes regulate the cell phenotype through changes in gene expression without altering the DNA sequence 99. Epigenetic drugs commonly target histone methyl-transferase, demethylases, and deacetylases to change transcriptomic profiles in order to treat tumors 100.

### 7.1. Histone deacetylase

Among kinds of epigenetic drugs against GBM, histone deacetylase (HDAC) inhibitors have drawn the most attention due to its broad mechanisms including cell-cycle arrest induction, differentiation, senescence, intrinsic and extrinsic apoptosis, mitotic cell death, autophagic cell death, inhibition of angiogenesis and metastasis, generation of reactive oxygen species, and enhancement in tumor immunity 101, 102. Several preclinical studies revealed HDAC inhibitors' potential to radio-sensitize cancers, including GBM 103. Currently, many clinical trials, mostly phase I and phase II are focusing on safety and efficacy profiles of HDAC inhibitors on GBM 104.

Vorinostat, the most advanced HDAC inhibitor, showed modest monotherapy activity (PFS=1.9 months, 6-month PFS=17%, median OS=5.7 months), while the combination of Vorinostat and other therapeutic agents is under research in multiple ongoing phase II trials 105. Notably, one recently completed phase I/II trial (NCT01266031) demonstrated that the median OS of Vorinostat in combination with Bevacizumab was shorter than that of Bevacizumab monotherapy (7.80 vs. 9.24 months).

Also, valproic acid (VPA), well known as an antiepileptic drug, is also an HDAC inhibitor, exhibiting impressive preclinical efficacy to radio-sensitize glioma cells 106. On the other hand, VPA also protects normal brain tissue and hippocampal neurons from radiotherapy 107. A phase II trial of VPA, TMZ, and concurrent radiotherapy for GBM patients presented promising results (median OS=29.6 months in newly diagnosed GBM patients) 108. Although promising, prospective data for VPA are still limited; to evaluate its efficacy and clarify optimal treating modality, further researches on VPA are in need^109^.

Other two HDAC inhibitors, Panobinostat and Romidepsin, both were proved to inhibit proliferation of GBM cells in vitro and in animal studies 110, 111. However, both of them showed disappointing results in phase II studies (NCT00859222 112, NCT00085540 113).

### 7.2. Histone methyltransferase and demethylase

As for the other two targets, studies on histone methyltransferase and demethylases have not made great achievement on GBM. Histone demethylases play a significant role in various malignant tumors, while its role in GBM is unclear 114. Azacytidine and Decitabine, two FDA-approved histone methyltransferase inhibitors 115, have not undergone clinically tests to evaluate its effect on GBM. Both histone methyltransferase and demethylases need to be illuminated in further details to develop as anti-GBM agents.

## 8. Oncolytic virus therapy

**Figure 4:**
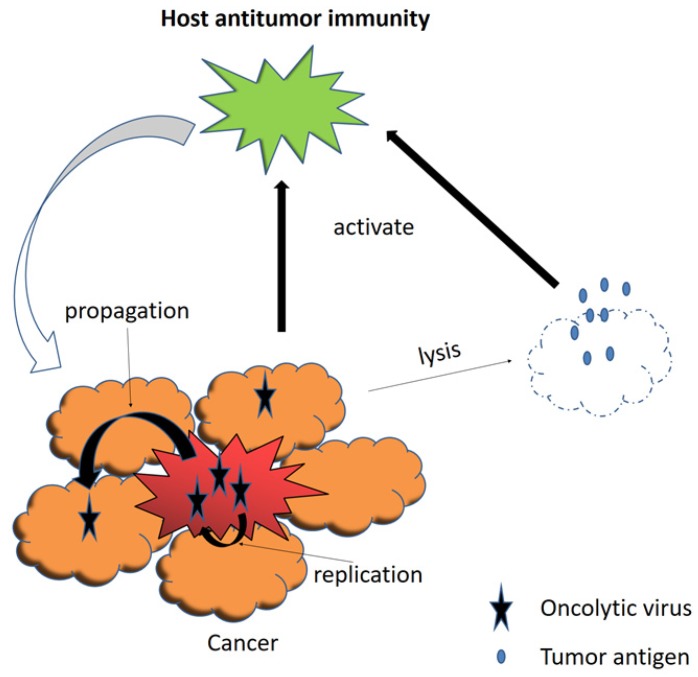
Viral infection and replication lyse tumor cells. The release of tumor-associated antigens induce systemic anti-tumor immunity.

An oncolytic virus, naturally occurring or genetically engineered, can selectively replicate in and kill cancer cells while sparing the normal ones 116. Viral infection and replication usually induce cellular stress, causing cell lysis 117, 118. Besides, oncolytic viruses can infect tumor vessel endothelium and inhibit tumor-related angiogenesis, resulting in additional cell death of tumor due to the lack of oxygen and nutrients 117, 119. It is worth to note that oncolytic virus therapy also remarkably induces systemic anti-tumor immunity as a result of the release of tumor-associated antigens, so as to prolong cancer patients' survival 120-122 (Fig. 4).

So far, two genetically engineered oncolytic viruses have been approved to be drugs: Oncorine for head/neck/esophagus cancer in China and T-Vec for melanoma. G47Δ, developed by Todo et al., is a promising third generation oncolytic HSV-1 to be tested in patients with recurrent or residual GBM in a phase II study (UMIN000015995) started in 2015 Japan 116.

The prospecting effect of oncolytic virus therapy has led to studies on many other engineered oncolytic viruses, including reovirus, ZIKV, parvovirus, poliovirus, vaccinia, and NDV (Newcastle disease virus).

One recent study suggests that ZIKV, an oncolytic virus, can preferentially target GSCs (glioblastoma stem cells) 123, possessing potential efficacy for GBM patients.

In a First Phase I/IIa Glioblastoma Trial, Oncolytic H-1 Parvovirus treatment showed a PFS of 15.9 weeks and an OS of 15.4 months 124.

Poliovirus infects cancer cells via binding CD-155, a cell adhesion molecule widely expressed in GBM 125. It causes the death of cancer cells and induces strong immunity against the tumor without severe side effects 126-129. These encouraging characteristics of poliovirus have led to an ongoing Phase I trial (NCT01491893) for recurrent GBM patients. The results demonstrated that PVSRIPO (polio-rhinovirus chimera) immunotherapy significantly improved the survival rate at 24 and 36 months compared with that of historical controls, with two patients alive more than 69 months 130.

Vaccinia and NDV (Newcastle disease virus) both are cytotoxic to GBM cells in vitro and induced tumor regression in vivo 131-134. However, they both lack satisfactory results from clinical trials.

Given the fact that oncolytic virus therapy is not yet an established approach to treat cancer, it may lead us to new paths if combined with immunotherapy or integrated with functional transgenes.

## 9. Gene therapy

**Figure 5:**
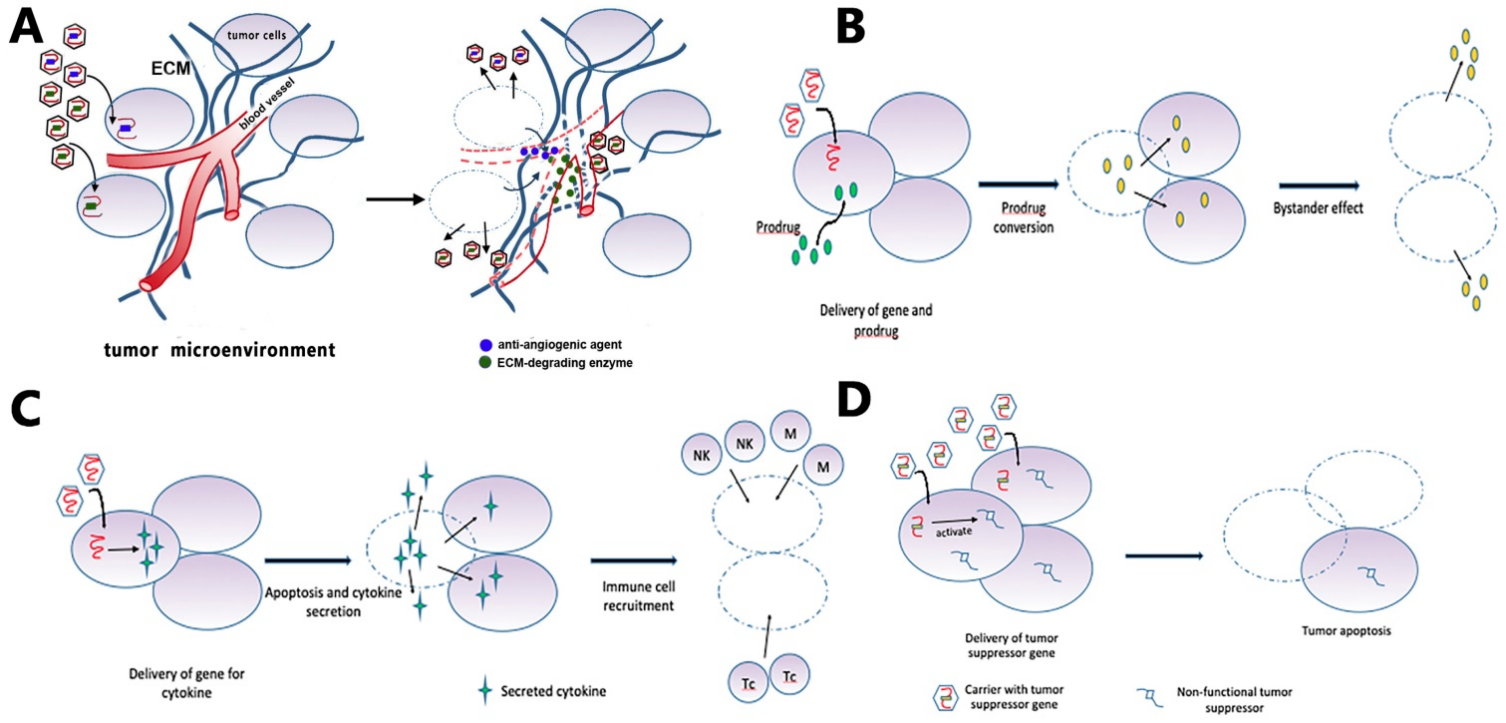
Gene therapy strategies. (A) Targeting the tumor microenvironment: viruses carry enzymes that degrade ECM components or anti-angiogenic factors that reduce vascular support of tumor. (B) GBM cells receive suicide genes from local injection of a carrier, together with systemic delivery of a prodrug. The suicide gene converts the prodrug into cytotoxic agents that kill the recipient cell and non-transduced bystander tumor cells. (C) The gene for an immunomodulatory cytokine is delivered to the tumor cells by viruses. Cytokine expression increases tumor cell apoptosis and activates immune cells such as macrophages, natural killer cells, and T-cell lymphocytes. (D) Tumor cells receive the functional copy of a tumor suppressor gene, which subsequently induces apoptosis.

Gene therapy is the treatment of disease through the introduction of therapeutic genes or manipulation of disease-related genes within target cells 135. Current gene therapy strategies for GBM can be roughly categorized into suicide gene therapy (Fig. 5B), tumor-suppressor gene therapy (Fig. 5D), oncolytic viral gene therapy, immune-modulatory gene therapy (Fig. 5C), and affecting the tumor microenvironment 136, 137 (Fig. 5A). The corresponding delivery systems can be categorized into three basic types: direct delivery (virus-mediated and non-virus mediated), tumor-tropic cell carriers, and other carriers aiming at the unique physiochemical environment of tumors 137.

### 9.1. Suicide gene therapy

Two well-studied suicide gene therapies are HSV (herpes simplex virus)-derived enzyme Thymidine Kinase (HSV-TK) and bacterial enzyme Cytosine Deaminase (CDA) 138-140.HSV-TK has been proved safe in multiple phase I and II clinical trials 141-143. However, the results from a phase III clinical trial for newly-diagnosed GBM patients were less exciting, with no significant improvements in PFS or OS 144.

CDA is currently under a phase I/II clinical trial (NCT01156584) for patients with recurrent high-grade glioma.

### 9.2. Tumor-suppressor Gene Therapy

p53 145, p16 146, p27 147, 148, and Phosphatase and Tensin Homologue (PTEN) 149 were proved effective to inhibit the growth and invasion of GBM cells. However, there have not been any promising clinical trials for them.

### 9.3. Immune-modulatory gene therapy

One excellent example was the expression of the gene for IFN-beta (interferon beta) in mice. In these animal studies, IFN-β resulted in potent immune responses against tumor and improved animal survival 150. A subsequent phase I trial examining IFN-β for recurrent malignant glioma demonstrated local inflammation and tumor necrosis 151. Other investigations on cytokines such as IL-12 and TNF-alpha have also received exciting results in animals 152, 153.

### 9.4. Affecting the tumor microenvironment

Researchers employed anti-angiogenic genes or genes that remodel the tumor extracellular matrix to manipulate the tumor microenvironment. The intra-tumor injection of anti-angiogenic factor angiostatin in some studies effectively inhibited tumor vascularization and tumor growth 154-156. Hence, anti-angiogenic gene therapy may hold future promise by affecting tumor microenvironment.

One study demonstrated increased efficacy of HSV against glioma when carried with chondroitinase ABC-I, a bacterial enzyme that degrades the glioma ECM (extracellular matrix). Surprisingly, degradation of ECM in this study did not lead to enhanced invasiveness of the remaining tumor cells 157.

Though promising in preclinical and phase I trials, anti-glioma gene therapies showed no significant benefits for patients in phase II and III trials 158-160. Notably, the blood-brain barrier has impeded intracranial tumor treatments for decades, while intracranial injection of vectors to circumvent blood-brain barrier could be an answer 158. Hence, besides exploring or optimizing gene therapy approaches, development of better viral or non-viral vectors to penetrate BBB, and precisely targeting tumor cells are both of great importance in order to treat GBM.

## Discussion

After many years of research, Glioblastoma, hiding behind the blood-brain barrier, continues to be the most devastating brain tumor. Due to its invasive nature, surgery alone can never cut out the whole lesion. Currently, radiotherapy and TMZ chemotherapy followed by surgery is the clinical standard, providing a mean survival time only about 14 to 16 months, not to mention the poor living quality of GBM patients. Although there had been a variety of treatment strategies under research, only TMZ and tumor treatment field were approved by FDA in the past 15 years.

To date, novel treatments including BNCT, anti-angiogenic therapy, immunotherapy, epigenetic therapy, oncolytic virus therapy, and gene therapy are still having either uncertain or discouraging clinical results. Reasons for the lack of progress in GBM treatment are many folds. First, the blood-brain barrier impedes most blood-borne drugs to target tumor cells. Although GBM always disturbs this barrier due to its malignancy, not all BBB near tumor cells is broken enough for drugs to penetrate. Second, GBM stem cells are responsible for tumor resistance to radiotherapy, and hence possibly many other novel therapies. Third, most of these clinical results derive from trials on recurrent GBM patients, so it is possible that recurred tumors are more refractory to any treatment, and hence the unsatisfying clinical trial results. Therefore, we might see more encouraging results from novel therapies if we could recruit newly diagnosed GBM patients though subject to ethical review. Also, in each treatment modality lies their own challenges. For example, in BNCT the challenge is to design more effective tumor-targeting boron compound.

So far, no monotherapy for GBM is enough. Developing novel therapies and exploring new combinations of therapies are the most challenging missions for physicians and researchers. For instance, Lomustine-temozolomide significantly improves the median OS versus temozolomide alone (48.1 vs.31.4 months) 24. Importantly, while exploring new combinations, a sound theoretical rationale should precede any random attempts.

Notably, many of the studies mentioned in this article have been conducted in vitro or in animals, requiring numerous subsequent clinical trials and tremendous effort to prove the feasibility and validity of studies. Though not satisfying to date, treatments for GBM shall have a brighter future with in-depth understanding of BBB, further understanding of tumor mechanisms, and the development of optimally combined treatment modalities.

## Figures and Tables

**Table 1:** Comparison of different treatment strategies. ND=not determined. N/A=not applicable.

	Categories	Target molecule	Clinical trial phase (Latest)	Survival advantage (OS mo.)	Other outcomes (complications)	Indication for other cancers (FDA approved)	Ref.
Chemotherapy	Temozolomide	DNA	FDA approved	2.5	Bone marrow suppression; nausea; emesis	Anaplastic astrocytoma	12-14
	BCNU	DNA crosslink	FDA approved	2.2	Bone marrow suppression; nausea; emesis	Medulloblastoma; astrocytoma; multiple myeloma	17-19
	Lomustine	DNA crosslink	phase III	16.7	Bone marrow suppression; nausea; emesis	lymphoma; gastric cancer	20-26
	Cyclophosphamide	DNA	phase II	ND	Bone marrow suppression; nausea; emesis	lymphoma; multiple myeloma; leukemia	27-31
Radiotherapy	N/A	DNA	FDA approved	5.4-7.7	Nausea; Emesis; Cognitive defect	Common type	32-51
TTF	N/A	Mitosis	FDA approved	2.8	topical skin rashes	ND	52-59
BNCT	N/A	GBM cell	phase II	2.2	ND	ND	60-64
Anti-angiogenic therapy	Bevacizumab	VEGF ligand	FDA approved	-0.4	Hypertension; thromboembolic	Colorectal cancer; Lung cancer; Renal cell cancer	65-71
	Nimotuzumab	EGFR	phase III	5.2	Chills; fever	Squamous carcinoma; Pancreatic cancer; Nasopharyngeal cancer	72-75
	ABT-414	EGFR	phase I	ND	Blurred vision; Keratitis	ND	76-77
Immunotherapy	Vaccination	EGFRvIII	phase III	2	ND	ND	78-84
	Adoptive cell therapy	EGFRvIII	phase II	5.9	ND	ND	85-86
	check-point inhibition	PD-1	phase II	6.2	Hypophysitis; encephalitis	Melanoma; lung/kidney cancer	87-91
	Immunostimulant	Immunity	phase II	6.4	toxicity	ND	92-98
Epigenetic therapy	Vorinostat(deacetylase)	Histone	phase II	-1.44	diarrhea	CTCL	101-105
	VPA ( deacetylase)		phase II	15	Nausea; emesis	ND	106-109
	Histone methyltransferase and demethylase	Histone	ND	ND	ND	ND	114-115
Oncolytic virus therapy	G47Δ	GBM cell	phase II	ND	ND	ND	116
	ZIKV	GBM cell	Pre-clinic	ND	ND	ND	123
	Oncolytic H-1 Parvovirus	GBM cell	phase II	ND	ND	ND	124
	Poliovirus	GBM cell	Phase I	ND	ND	ND	125-130
	Vaccinia	GBM cell	Pre-clinic	ND	ND	ND	131-134
	NDV	GBM cell	Pre-clinic	ND	ND	ND	131-134
Gene therapy	Suicide gene therapy	gene	phase II	0.4	ND	ND	138-144
	Tumor-suppressor Gene Therapy	gene	Pre-clinic	ND	ND	ND	145-149
	Immune-modulatory gene therapy	gene	Pre-clinic	ND	local inflammation	ND	150-153
	Affecting the tumor microenvironment	gene	Pre-clinic	ND	ND	ND	154-157
